# Interpreting negative test results when assessing cancer risk in general practice

**DOI:** 10.3399/bjgp21X716189

**Published:** 2021-06-25

**Authors:** Stephen H Bradley, Brian D Nicholson, Garth Funston

**Affiliations:** Leeds Institute of Health Sciences, University of Leeds, Leeds.; Nuffield Department of Primary Care Health Sciences, University of Oxford, Oxford.; Primary Care Unit, Department of Public Health and Primary Care, University of Cambridge, Cambridge.

Studies published over the last year have established the sensitivity of chest X-ray (CXR) for lung cancer (75%, 95% confidence interval [CI] = 68 to 83), cancer antigen 125 (CA125) for ovarian cancer (77%, 95% CI = 73 to 81), and the faecal immunochemical test (FIT) for colorectal cancer (91%, 95% CI = 85 to 96) in symptomatic people attending primary care.^1^^–^^3^ This research demonstrates how simple and accessible tests can be used by GPs to identify these cancers in most cases; however, it also raises questions about how GPs should respond to negative test results in situations in which there is some concern about the possibility for cancer, but criteria for an urgent suspected cancer referral are not met.

## THE FALSE-NEGATIVE DILEMMA

With sensitivities of 75%–91%, specificities of 90%–94%, and negative predictive values exceeding 99%, individual patients can take a good deal of reassurance from a negative CXR, CA125, or FIT;^1^^–^^3^ however, it is important to recognise that the high negative predictive values of these tests are a function of the low prevalence of cancer in primary care (there are many more true-negative than false-negative results).

Patients tested in primary care who have a negative CXR, CA125, or FIT are therefore very unlikely to have lung, ovarian, or colorectal cancer, respectively. But, the imperfect sensitivity of these tests means that around one in five to one in ten patients who do have cancer will have a negative test.

A full-time equivalent GP would be expected to encounter roughly one case of lung cancer every year and, given the test’s sensitivity, one case on average would not be detected by CXR every 5 years.^4^^–^^5^ Onward referral of all patients who have possible symptoms of cancer but negative primary care tests is neither appropriate nor feasible. Approximately 330, 500, and 1000 primary care patients with negative CXRs, CA125, and FIT, respectively, would have to undergo further investigation in order to detect one additional cancer case.^1^^–^^3^ The limitations of these tests mean they should only ever be a complement to, rather than a substitute for, the skilled interpretation of clinical information and the application of professional judgement, regarding the underlying risk of cancer.

## PRE- AND POST-TEST PROBABILITY

The risk (or pre-test probability) of cancer in patients attending primary care is low. A positive or negative test result leads to an increased or decreased risk (post-test probability). Negative test results for CXR, CA125, and FIT make cancer diagnoses much less likely, but by no means impossible. The extent of the residual risk hinges on the pre-test probability of cancer. The predictive power of the clinical history should sometimes trump test results. For example, unexplained haemoptysis is so strongly associated with lung cancer that immediate referral is justified without waiting for CXR, as the risk of cancer even following a negative CXR is 3%.^3^ Although clear cut red flag symptoms like haemoptysis are usually not present, many cues may emerge in the consultation that inform an assessment of the probability that a serious disease such as cancer is present.^6^ GPs should carefully gauge their index of concern and use this to inform decision making before and after receiving test results.^7^

## NOT ALL ‘NORMAL’ RESULTS ARE EQUAL

Age strongly affects the pre-test probability, and therefore also the post-test probability, of cancer. For example, the estimated risk of ovarian cancer in a 70-year-old female with a CA125 of 34 U/ml (just below the national cut-off) is 3.3% (95% CI = 2.6 to 4.1), while for a 40-year-old female with the same CA125 level the risk is 0.9% (95% CI = 0.6 to 1.2).^1^ For cancer tests measured on a continuous scale, such as CA125 and FIT, it may be useful to consider the test level when interpreting the result, not just whether the test is categorised as ‘normal’ or ‘abnormal’.^1^^,^^8^ A ‘borderline’ result (just below the test cut-off) indicates a greater risk of cancer than a very low test value and, when considered in conjunction with other clinical information, this could help patients and clinicians make individualised decisions about follow-up.

## HARNESSING ‘GUT FEELING’

Symptoms and signs are only a fragment of the clinical information collected during the consultation. A GP’s ‘gut feeling’ represents the rapid summing up of verbal and nonverbal cues in the context of the GP’s clinical knowledge and experience.^9^ GPs have reported relying on gut-feelings when caring for patients whose presentation falls into the ‘grey area’ of primary care practice, where clinical guidance does not adequately address the patient’s presentation, such as patients with negative test results. Though this intuition is neither clairvoyant nor precise, it is one more tool that GPs can harness alongside knowledge of guidelines, evidence, and an understanding of test interpretation. Understanding test performance and triggers of GP gut feelings are therefore crucial considerations when developing safety-netting strategies for people who have negative test results.^10^

## SAFETY NETTING

When faced with negative test results, we should consider the presentation that informed the decision to test. Even if the post-test probability is very low, any reassurance must be limited if testing was informed by a general sense that ‘something is wrong’, rather than a strong suspicion for one particular disease.

Although patients may give their reason for re-attendance as ‘getting their results’, the clinical challenge is not simply to relay the result, but to agree a suitable plan that is informed both by the test and an assessment of the probability of serious pathology. GPs who are following up on tests that have been requested by colleagues should take care to understand the reasons for investigation and expect to undertake a re-evaluation of the patient’s present condition.

Appropriate management of negative test results will depend largely on an assessment of clinical risk, but may include:

- reassessment for new or evolving symptoms and to obtain a fresh perspective of risk;
- obtaining advice from colleagues or specialists when there is doubt as to whether urgent referral is warranted;
- repeating the test, after an appropriate interval;
- investigation with other direct access modalities such as endoscopy, computed tomography, or ultrasound, if available;
- urgent suspected cancer referral, justified by symptoms or GP concern (gut feeling), where permitted by local pathways;
- referral to multidisciplinary diagnostic centres if insufficient localising symptoms for specific pathway but still significant concern, if available;
- a plan negotiated with the patient outlining circumstances such as a persistence of symptoms within a timeframe, or evolution of symptoms that should prompt reconsultation; and
- reassurance, where, based on the test result and clinical scenario, the risk of cancer is deemed as very low.

## SHARE THE BURDEN OF UNCERTAINTY

The importance of achieving timely cancer diagnosis and the need to steward resources responsibly in publicly-funded healthcare systems places considerable pressure on GPs. It is probably inevitable that GPs will sometimes be criticised in retrospect for following conservative management strategies, such as safety netting, in preference to referral, in patients deemed to be at low risk of cancer at presentation but who are subsequently diagnosed with cancer.^11^ However, an understanding of the performance of tests used in cancer diagnosis can help inform judgements of the extent of risk that remains following a negative result. Unfortunately, we lack a comprehensive understanding of how some important tests, such as prostate specific antigen, perform in primary care populations.

High degrees of uncertainty connote a broad range of possible management decisions. Patients interpret risk differently and the thresholds of risk that are acceptable vary from patient to patient, and these are likely to differ from those of their GPs.^12^ We should feel able to acknowledge uncertainty and share decision making with patients. Where risk is deemed to be low, but not absent, safety netting is a well-established means of sharing responsibility, but we need to outline advice in clear terms, with stated reasons for re-presentation and well-defined time scales where possible.^13^

Timely cancer diagnosis and managing uncertainty are core functions of primary care. Knowledge of the predictive power of symptoms and of common test results are important tools to help GPs identify cancer early. While recent studies have demonstrated that currently available cancer tests are valuable tools and perform well for the detection of cancer in primary care,^1^^–^^3^ such tests are (and always will be) imperfect. The vast majority of patients with a negative test result will not have cancer — identifying those who do is a major diagnostic challenge. However, combining an understanding of test performance with clinical judgement and patient preference should help select appropriate follow-up strategies for patients with negative results and could contribute to timely cancer detection.
